# The Effect of Race and Social Vulnerability on the Management of Thumb Carpometacarpal Osteoarthritis

**DOI:** 10.7759/cureus.78939

**Published:** 2025-02-13

**Authors:** Andrea H Johnson, Jane C Brennan, Faith I Wheeler, Parimal Rana, Justin J Turcotte, Risa Reid

**Affiliations:** 1 Orthopedics, Anne Arundel Medical Center, Annapolis, USA; 2 Orthopedic Research, Anne Arundel Medical Center, Annapolis, USA; 3 Medicine, HCA Florida Lawnwood Hospital, Fort Pierce, USA; 4 Orthopedic and Surgical Research, Anne Arundel Medical Center, Annapolis, USA; 5 Orthopedic Surgery, Anne Arundel Medical Center, Annapolis, USA

**Keywords:** carpometacarpal (cmc) joints, cmc joint arthroplasty, non-operative treatment, racial disparities, social vulnerability index, socioeconomic disparities, thumb carpometacarpal arthritis

## Abstract

Introduction

Thumb carpometacarpal (CMC) osteoarthritis is one of the most common hand arthropathies. There is significant variability in treatment, and understanding how race and social vulnerability impact treatment decisions is essential for equitable care. The purpose of this study is to examine the effect of race and social vulnerability on the management of patients with thumb CMC osteoarthritis.

Methods

A retrospective review of 270 patients presenting to one community-based health system for CMC osteoarthritis from December 2014 to February 2023 was performed. Patient demographics, comorbidities, patient-reported outcomes, social vulnerability index (SVI), and Eaton-Littler classification were collected. Patients were classified by race and SVI. The primary outcome of interest was CMC arthroplasty. Secondary outcomes included non-operative treatment and time to surgery.

Results

On average, non-White patients were younger (p=0.033), had increased BMI (p=0.001), and were more likely females (p=0.002). Additionally, non-White patients were more socially vulnerable overall than White patients (p<0.001). Non-White patients had a higher rate of steroid injection (p=0.018), a lower rate of splinting (p<0.001), and a lower rate of CMC arthroplasty (21.5% vs. 35.6%; p=0.038). On multivariate analysis, non-White patients were 2.17 (p=0.035) times less likely to have CMC arthroplasty than White patients.

Conclusions

Non-White and higher social vulnerability patients are less likely to receive a splint and proceed to CMC arthroplasty. On multivariate analysis, the White race patients were predictive of CMC arthroplasty. On multivariate analysis, White race patients were associated with increased odds of CMC arthroplasty. These findings highlight the association between socioeconomic and racial factors and treatment decisions, suggesting a need for targeted strategies to ensure equitable care.

## Introduction

Thumb carpometacarpal (CMC) osteoarthritis is one of the most common locations for degenerative arthritis of the hand and is present in a significant percentage of the population [1-3]. The incidence of this condition rises with age, affecting over 90% of individuals aged 80 and above [3-5]. Thumb CMC osteoarthritis can be a significant source of pain and disability, hindering function and affecting quality of life; despite this, relatively few patients seek treatment for this condition [2,6-8]. The mainstays of nonoperative treatment of CMC arthritis are similar to the treatment of osteoarthritis in other joints and include activity modification, therapy, nonsteroidal anti-inflammatory drugs (NSAIDs), intraarticular injections with cortisone or another medication, and splinting [5,9,10]. While most patients can be adequately treated with nonoperative measures, surgical interventions are available. The most common surgical procedure for CMC osteoarthritis is a resection arthroplasty, although arthrodesis and arthroplasty with a prosthesis are also available options [5,11].

There is a debate among providers as to what is the most appropriate management of CMC osteoarthritis [7,12,13]. There is considerable variation among patients in their decisions to seek treatment, continue with nonoperative management, or opt for surgical intervention [8,14,15]. Identifying factors that contribute to patients seeking specific types of treatment is important, as studies have shown that radiographic severity of disease is not predictive of treatment patterns [2,8,16]. Racial and socioeconomic factors have been shown to affect utilization, treatment patterns, and patient outcomes in orthopedic surgery. However, a significant amount of the research has focused on larger and more costly procedures such as joint replacement and spine surgery [17-21]. In elective hand surgery, research on disparities in utilization and outcomes has been relatively limited, with no studies specifically examining thumb CMC osteoarthritis. The purpose of this study is to examine the association of race and social vulnerability with treatment utilization in patients presenting to an orthopedic hand clinic with thumb CMC osteoarthritis. We hypothesize that non-White and more socially vulnerable patients will have decreased utilization of non-operative treatments and CMC arthroplasty.

## Materials and methods

Study population

This study was deemed exempt by the Institutional Review Board of Luminis Health Research Institute. A retrospective cohort study of 270 patients presenting to a hand surgeon at one community-based health system for thumb CMC osteoarthritis from March 2021 to February 2023 was performed. Patient records from July 2014 to February 2023 were reviewed to evaluate historical visits for CMC osteoarthritis. Patients were treated by one of five fellowship-trained orthopedic hand surgeons in an ambulatory orthopedic clinic.

Outcome measures

The primary outcome of interest was whether the patient experienced surgical intervention with CMC arthroplasty. Secondary outcomes included the number of steroid injections administered, splinting as a form of treatment, and the time taken for surgery.

Independent variables

The independent variables of interest included age, body mass index (BMI), ethnicity, smoking status, comorbidities including history of trauma or injury, history of rheumatoid arthritis, history of carpal tunnel syndrome, Ehlers-Danlos syndrome, diabetes, baseline quick disabilities of the arm, shoulder, and hand (QuickDASH) score, the centers for disease control agency for toxic substances and disease registry (CDC/ATSDR) social vulnerability index (SVI), presenting Eaton-Littler classification and final preoperative or last available Eaton-Littler classification.

Social vulnerability index

The CDC/ATSDR SVI is a database that was initially created to identify populations at risk before, during, or after emergency situations such as weather disasters or disease outbreaks [22]. The database uses 16 census variables and groups them into four themes: socioeconomic status, household characteristics, racial and ethnic minority status, and housing type and transportation. Each subdivision receives a separate ranking for each theme; more vulnerable communities have a higher score.

Statistical analysis

Patients were classified by race and social vulnerability. We chose to divide race into White and non-White due to the sample size and race distribution. Of the 270 patients, 191 (70.7%) were White patients, and 79 (29.3%) were non-White patients; 59 (74.7%) of the non-White patients were of Black race, with the remaining 20 (25.3%) distributed between Asian, Native American, two or more races, or other. The SVI was used to quantify socioeconomic disadvantage based on patients’ zip code of residence. Overall, SVI scores were evaluated. Higher scores on the SVI represent greater levels of social vulnerability. Patients with an overall SVI greater than 0.5 were considered high-vulnerability, while patients with an overall SVI less than or equal to 0.5 were considered low vulnerability. There were 240 patients with trackable zip codes.

Univariate analysis, including chi-square tests, Fisher exact tests, and two-sided independent samples t-tests, were used to determine differences in patient characteristics and outcomes between White and non-White patients and high and low-vulnerability patients. Multivariate logistic regression was used to determine factors associated with CMC arthroplasty. Only factors that were significant in the univariate analysis comparing White and non-White patients were included in the multivariate model. All statistical analyses were performed using R Studio (Version 4.2.2 © 2009-2024 RStudio, PBC). Statistical significance was assessed at p<0.05.

## Results

On average, non-White patients were younger (60.8±11.0 years vs. 63.9±9.4 years; p=0.033), had increased BMI (32.1±7.4 kg/m^2^ vs. 28.8±6.0 kg/m^2^; p=0.001) and were more likely female (88.6% vs. 69.6%; p=0.002) compared to White patients. Additionally, non-White patients were more socially vulnerable overall than White patients (0.44±0.22 vs. 0.27±0.19; p<0.001). There were no differences in ethnicity, smoking status, comorbidities, baseline QuickDASH, initial Eaton-Littler classification, and final Eaton-Littler classification between White and non-White patients (Table 1). 

**Table 1:** Patient characteristics by race. All data is presented as n(%) or mean±SD; p<0.05 in bold; *indicates Fisher’s exact test; t-score: two-sided t-test; X^2^: chi-square test; SVI: social vulnerability index; QuickDASH: quick disabilities of the arm, shoulder, and hand.

Patient characteristics	White (n=191)	Non-White (n=79)	Test statistic	p-value
Age, years	63.9±9.4	60.8±11.0	t-score=2.156	0.033
Body mass index, kg/m^2^	28.8±6.0	32.1±7.4	t-score=-3.390	0.001
Sex			Χ^2^=9.791	
Female	133 (69.6)	70 (88.6)		0.002
Male	58 (30.4)	9 (11.4)		
Hispanic ethnicity	6 (3.1)	6 (7.6)		0.116*
Smoking status				0.430*
Former	61 (31.9)	22 (27.8)		
Current	14 (7.3)	3 (3.8)		
Never	116 (60.7)	54 (68.4)		
Trauma/injury	30 (15.7)	10 (12.7)	X^2^ = 0.205	0.650
Rheumatoid arthritis	6 (3.1)	3 (3.8)		0.724*
Carpal tunnel syndrome	61 (31.9)	23 (29.1)	X^2^=0.097	0.756
Ehlers-Danlos syndrome	1 (0.5)	0 (0)		1*
Diabetes mellitus	24 (12.6)	14 (17.7)	X^2^=0.839	0.360
Overall SVI score	0.27±0.19	0.44±0.22	t-score=-5.894	<0.001
Baseline QuickDASH	41.9±19.3	50.5±25.0	X^2^=-1.531	0.138
Initial Eaton-Littler classification				0.735*
1	15 (8.3)	4 (7.3)		
2	45 (25.0)	12 (21.8)		
3	103 (57.2)	36 (65.5)		
4	17 (9.2)	3 (5.5)		
Final Eaton-Littler classification				0.902*
1	0 (0)	0 (0)		
2	9 (12.7)	3 (18.8)		
3	52 (73.2)	11 (68.8)		
4	10 (14.1)	2 (12.5)		

Non-White patients had a higher rate of any steroid injection (70.9% vs. 54.5%; p=0.018), a higher rate of multiple steroid injections (50.6% vs. 35.1%; p=0.025), a lower rate of therapeutic splinting (72.4% vs. 91.6%; p<0.001) and a lower rate of CMC arthroplasty (21.5% vs. 35.6%; p=0.034) compared to White patients (Table 2). 

**Table 2:** Outcomes by race. All data is presented as n(%) or mean±SD; p<0.05 in bold; t-score: two-sided t-test; X^2^: chi-square test; CMC: carpometacarpal.

Outcome	White (n=191)	Non-White (n=79)	Test statistic	p-value
Initial conservative treatment	171 (89.5)	75 (94.9)	X^2^=1.406	0.236
Received any steroid injection	104 (54.5)	56 (70.9)	X^2^=5.591	0.018
Received multiple steroid injections	67 (35.1)	40 (50.6)	X^2^=5.020	0.025
Therapeutic splint	175 (91.6)	58 (72.4)	X^2^=14.161	<0.001
CMC arthroplasty	68 (35.6)	17 (21.5)	X^2^=4.506	0.034
Time to surgery, months	14.6±15.5	22.6±26.0	t-score=-1.229	0.234

Of the 240 patients with an SVI, (194) 80.8% were low vulnerability, and 46 (19.2%) were high-vulnerability. The high-vulnerability group had a greater percentage of non-White patients (60.9% vs. 24.2%; p<0.001) compared to the low-vulnerability group. Additionally, there was a significant difference in the distribution of initial Eaton-Littler classification between high and low-vulnerability groups. Low-vulnerability had 9.0%, 28.7%, 55.6%, and 6.7% of patients with grades one, two, three, and four, respectively, while high-vulnerability patients had 3.3%, 6.7%, 80.0%, and 10.0% of patients with grades one, two, three, and four respectively (p=0.017) (Table 3). 

**Table 3:** Patient characteristics by social vulnerability. All data is presented as n(%) or mean±SD; p<0.05 in bold; *indicates Fisher’s exact test; t-score: two-sided t-test; X^2^​​​​​: chi-square test; QuickDASH: quick disabilities of the arm, shoulder, and hand.

Patient characteristics	Low vulnerability (n=194)	High vulnerability (n=46)	Test statistic	p-value
Age, years	63.1±10.0	62.5±10.6	t-score=-2.785	0.708
Non-White race	47 (24.2)	28 (60.9)	X^2^=21.564	<0.001
Body mass index, kg/m^2^	29.2±6.3	31.1±6.6	t-score=-4.091	0.092
Sex			X^2^=0.682	0.409
Female	142 (73.2)	37 (80.4)		
Male	52 (26.8)	9 (19.6)		
Hispanic ethnicity	8 (4.1)	3 (6.5)		0.445*
Smoking status				1*
Former	60 (30.9)	14 (30.4)		
Current	13 (6.7)	3 (6.5)		
Never	121 (62.4)	29 (63.0)		
Trauma/injury	29 (14.9)	6 (13.0)	X^2^=0.009	0.923
Rheumatoid arthritis	4 (2.1)	2 (4.3)		0.324*
Carpal tunnel syndrome	61 (31.4)	12 (26.1)	X^2^=0.283	0.595
Ehlers-Danlos syndrome	0 (0)	0 (0)		1*
Diabetes mellitus	26 (13.4)	8 (17.4)	X^2^=0.214	0.644
Baseline QuickDash	42.0±20.2	49.8±26.8	t-score = -27.890	0.371
Initial Eaton-Littler classification				0.017*
1	16 (9.0)	1 (3.3)		
2	51 (28.7)	2 (6.7)		
3	99 (55.6)	24 (80.0)		
4	12 (6.7)	3 (10.0)		
Final Eaton-Littler classification				0.829*
1	0 (0)	0 (0)		
2	10 (14.1)	1 (12.5)		
3	52 (73.2)	7 (87.5)		
4	9 (12.7)	0 (0)		

High-vulnerability patients had a lower rate of therapeutic splinting (76.1% vs. 88.7%; p=0.047) compared to low-vulnerability patients. There were no differences in conservative treatment, steroid injections, rates of CMC arthroplasty, or time to surgery between groups (Table 4). 

**Table 4:** Outcomes by social vulnerability score. All data presented as n (%) or mean±SD; p < 0.05 in bold; *indicates Fisher’s exact test; t-score indicates two-sided t-test; X^2^ indicates chi-square test; CMC: carpometacarpal.

Outcome	Low vulnerability (n=194)	High vulnerability(n=46)	Test statistic	p-value
Initial conservative treatment	179 (92.3)	42 (91.3)		1*
Received any steroid injection	112 (57.7)	34 (73.9)	X^2^=3.435	0.064
Received multiple steroid injections	74 (38.1)	24 (52.2)	X^2^=2.477	0.116
Therapeutic splint	172 (88.7)	35 (76.1)	X^2^=3.953	0.047
CMC arthroplasty	62 (32.0)	12 (26.1)	X^2^=0.357	0.550
Time to surgery, months	17.3±8.8	9.5±12.6	t-score=-1.211	0.086

After controlling for age, BMI, sex, and high-vulnerability, non-White patients were 2.17 (OR: 0.46, 95% CI: 0.21 to 0.93; p=0.035) times less likely to undergo CMC arthroplasty compared to White patients (Table 5). In this model, 17.8% of the variability in who proceeds with CMC arthroplasty is accounted for by the factors included in our model.

**Table 5:** Predictors of CMC arthroplasty. p<0.05 in bold; R^2^=0.178

Predictor	Estimate	Odds ratio	95% CI	Z-value	p-value
Age	0.004	1.00	0.98 to 1.03	0.293	0.770
Body mass index	-0.013	0.99	0.94 to 1.03	-0.557	0.577
Male	-0.175	0.84	0.43 to 1.59	-0.527	0.598
Non-White race	-0.783	0.46	0.21 to 0.93	-2.111	0.035
High-vulnerability	0.075	1.08	0.48 to 2.33	0.190	0.850

## Discussion

In this study, while non-White patients had increased social vulnerability when compared with White patients, they presented with similar comorbidities and at a similar Eaton-Littler classification; they were, on average, three years younger and had a higher BMI. When looking at treatment for thumb CMC osteoarthritis, non-White patients had a higher rate of steroid injections, although they were less likely to proceed to CMC arthroplasty when compared to White patients. Nearly 20% of patients in this study were classified as high social vulnerability. Patients in this group were more likely to be non-White and had a higher Eaton-Littler classification on initial presentation, although there were no differences in treatment other than a lower rate of therapeutic splinting. On multivariate analysis, non-White patients were significantly less likely to have CMC arthroplasty.

Many patients with symptomatic thumb CMC osteoarthritis can be managed with conservative treatment, including NSAIDs, intraarticular joint injections, and therapeutic splinting [8,9]. CMC joint injections, most commonly with corticosteroids, have been shown to provide significant long-term relief of symptoms, particularly in earlier-stage disease [9,23]. Therapeutic splinting, with a variety of orthoses, including custom-made and off-the-shelf varieties, has also been shown to provide long-term relief of symptoms and functional improvement [9,10]. In this study, more than 60% of patients received at least one CMC joint corticosteroid injection, and more than 80% of patients received a splint. However, non-White patients were more likely to receive an injection, while White and less vulnerable patients were more likely to receive a splint.

When symptoms are not adequately managed with conservative treatment, surgical intervention may be indicated. The most common surgery for thumb CMC osteoarthritis is a resection arthroplasty [5]. Few studies have examined which patient factors may contribute to an increased likelihood of surgical intervention as well as the time from treatment initiation to surgery. A study by Schloemann et al. of 1,994 patients found that only 9% of patients underwent surgery for CMC osteoarthritis, and the surgery occurred at a mean of four months after the first visit; contralateral thumb CMC osteoarthritis surgery and surgeon preference were the only predicting factors, whereas pain and functional limitations were not predictive [8]. Ostergaard et al. examined 239 patients who had a first-time corticosteroid injection for thumb CMC osteoarthritis and found that more than one-third of these patients underwent surgery within five years and that advanced radiographic osteoarthritis, current smoking, and history of ipsilateral hand surgery were predictive of undergoing surgery [15]. In our study, nearly one-third of patients underwent CMC arthroplasty at a mean of just over one year after initial presentation. The only factor we identified as associated with CMC arthroplasty was the White race.

Racial disparities are known to contribute to poorer surgical outcomes in patients undergoing elective orthopedic procedures for arthritic conditions of the upper extremity [17,24]. A number of studies have also identified racial disparities in the utilization of upper extremity surgery; Quinn et al. found that non-White patients were less likely to undergo rotator cuff repair after rotator cuff injury [25]. Similarly, two separate studies by Brodeur et al. found non-White patients were less likely to undergo carpal tunnel release and trigger finger release [26,27]. In a study of 361,942 African-American and White patients with a diagnosis of carpal tunnel syndrome, Hooper et al. found that African-American patients were significantly less likely to choose surgery as their first option, and this gap increased over the 11 years of the study [28]. Similarly, non-White patients in our study had a lower rate of surgery when compared with White patients. This difference was also present in the multivariate analysis, with White race being associated with increased odds of surgery.

The disparities in surgical utilization between White and non-White patients present a significant issue as health systems strive to improve health equity and equality benefits for all. A 2021 study by Best et al. of nine common surgical procedures found that over a five-year period, the disparity in surgical utilization either persisted or worsened between Black and White patients despite national initiatives aimed at addressing this problem [29]. Unconscious or implicit bias has been identified as a factor in disparities of access and outcomes [30-32]. Ibrahim et al. found that Black patients and White patients had different expectations regarding pain, hospital course, and recovery following joint replacement and that these factors influenced Black patients' willingness to undergo surgery [33]. Additionally, mistrust of medical institutions and providers also plays a significant role in ongoing disparities in healthcare utilization and outcomes [34,35]. While these factors are challenging to address, both individually and as a whole, increased awareness of the issues will help to improve ongoing racial disparities.

The contributions of social vulnerability to healthcare outcomes and disparities are perhaps somewhat more nuanced than racial disparities, although it is still an important factor to consider. Increased social vulnerability is associated with poorer clinical outcomes in upper extremity surgery [36-38]. A number of studies have also implicated social vulnerability as a factor in undergoing surgery. Kerluku et al. found that patients with a higher social deprivation index were less likely to be recommended for carpal tunnel release and were also less likely to undergo surgery [39]. Brodeur et al. identified similar findings in patients undergoing trigger finger release, with more socially vulnerable patients being less likely to undergo surgery [27]. Quinn et al. found that more socially vulnerable patients were less likely to undergo rotator cuff repair following a rotator cuff tear [25]. In contrast, in this study, patients with high social vulnerability (SVI>0.5) had surgery at the same rate as patients with low social vulnerability, although the high-vulnerability patients were less likely to undergo splinting prior to surgery. High social vulnerability patients also tended to present with a higher Eaton-Littler classification when compared to lower vulnerability patients, which may indicate a lack of access to care or the need to delay care due to other social factors.

The results of this study need to be considered in light of its limitations. As a retrospective study from a single institution, the results may not be representative of the wider population of patients with thumb CMC osteoarthritis, and selection bias may exist. In addition, this study draws from a limited geographic area, primarily suburban in nature, where there may be less variation in social vulnerability than in other geographic areas. However, the racial breakdown of 70% White and 30% non-White is similar to that of the United States population as a whole [40]. The multivariate analysis included in this study had a low R^2 ^value, reflecting the complex nature of surgical decision-making. Given that the model only explained approximately 18% of the variation in progression to surgery, there are likely many other factors influencing the decision to undergo surgery that warrant further investigation.

Additionally, the aim of this model was to examine associations between individual patient factors and surgery utilization rather than to develop a comprehensive model for predicting which patients proceed to surgery. Moreover, this study did not include information regarding patient satisfaction or functional abilities following surgical or non-surgical treatment, which would contribute significantly to this discussion. Ultimately, the decision to treat CMC osteoarthritis is dependent on patient and provider preference, as the surgeon’s attitude towards CMC osteoarthritis treatment has been shown to influence treatment patterns [8]. Despite these limitations, we feel that this study is a valuable contribution to the literature as it is one of the few studies that examine the association of race and social vulnerability with treatment patterns in thumb CMC osteoarthritis.

## Conclusions

For patients presenting to a single ambulatory surgical clinic with thumb CMC osteoarthritis, non-White and higher social vulnerability patients are less likely to receive a splint and proceed to thumb CMC arthroplasty. In addition, non-White patients were more likely to receive one or more corticosteroid injections. Arthritis grade by Eaton-Littler classification was similar between White and non-White patients at initial presentation and final radiograph; patients with higher social vulnerability had higher graded arthritis at initial presentation. On multivariate analysis, White race was associated with increased odds of CMC arthroplasty. This study highlights the association of socioeconomic and racial factors with treatment interventions in patients with thumb CMC osteoarthritis and thus emphasizes the need for further investigation on additional factors that influence the decision for thumb CMC osteoarthritis surgery as well as the implications for patients choosing surgical versus non-surgical treatment.
